# Differential toxicity to murine small and large intestinal epithelium induced by oncology drugs

**DOI:** 10.1038/s42003-022-03048-x

**Published:** 2022-01-27

**Authors:** Jake M. Bieber, Laura E. Sanman, Xiaoxiao Sun, Heinz Hammerlindl, Feng Bao, Maike A. Roth, Megan L. Koleske, Liusheng Huang, Fran Aweeka, Lani F. Wu, Steven J. Altschuler

**Affiliations:** 1 Department of Pharmaceutical Chemistry, University of California, San Francisco, San Francisco, CA 94158 USA; 2 Graduate Program in Bioengineering, University of California, San Francisco and University of California, Berkeley, San Francisco, CA 94158 USA; 3 Department of Bioengineering and Therapeutics, University of California, San Francisco, San Francisco, CA 94158 USA; 4 Drug Research Unit, Department of Clinical Pharmacology, University of California, San Francisco, San Francisco, CA 94158 USA

**Keywords:** Growth factor signalling, Gastroenterology, Gastrointestinal models, Transporters, Toxicology

## Abstract

Gastrointestinal toxicity is a major concern in the development of drugs. Here, we establish the ability to use murine small and large intestine-derived monolayers to screen drugs for toxicity. As a proof-of-concept, we applied this system to assess gastrointestinal toxicity of ~50 clinically used oncology drugs, encompassing diverse mechanisms of action. Nearly all tested drugs had a deleterious effect on the gut, with increased sensitivity in the small intestine. The identification of differential toxicity between the small and large intestine enabled us to pinpoint differences in drug uptake (antifolates), drug metabolism (cyclophosphamide) and cell signaling (EGFR inhibitors) across the gut. These results highlight an under-appreciated distinction between small and large intestine toxicity and suggest distinct tissue properties important for modulating drug-induced gastrointestinal toxicity. The ability to accurately predict where and how drugs affect the murine gut will accelerate preclinical drug development.

## Abstract (web summary)

Bieber et al use intestinal monolayers derived from fresh murine crypts as a model system to study drug toxicity. Testing 48 oncology drugs with known gastrointestinal toxicity as a proof of concept, they discover differential responses in cells from large and small intestine, increasing our mechanistic understanding of intestinal drug toxicity.

## Introduction

The leading cause of attrition in drug development is toxicity^1,2^. Drug-induced toxicity is first assessed through acute toxicology studies in animals, which are conducted as a precursor to clinical trials^3^. By this stage, much effort has already been expended in drug candidate optimization, and drug failures are extremely costly. Animal studies are low-throughput and expensive, and it is currently infeasible to screen compounds for toxicity earlier in development when there are hundreds of candidates. Development of scalable models for predicting drug-induced toxicity can guide decisions early in drug development, reduce pre-clinical failures and enable the progression of safer drug candidates^4^.

The gastrointestinal (GI) tract is one of the most common sites of toxicity both in drug development and in the clinic^2,5,6^. Several in vitro intestinal models have been recently developed that enable systematic investigation of intestinal drug absorption, drug metabolism, and anticancer efficacy^7–9^. These systems either utilize cancer-derived cells, lack both proliferative and differentiated cell types, or do not model both the small and large intestines. Current evaluation of drug-induced GI toxicity continues to rely heavily on histological analysis of rodent intestinal epithelia. These studies describe the types of drug-induced damage to the intestinal epithelium, including villus stunting, crypt dysplasia, and mucin hypersecretion^10–12^, yet it remains a challenge to pinpoint biological mechanisms of GI toxicity.

Further, there are differences in the physiology and biology between the small and large intestine which may cause drugs to target parts of the GI tract differently. For example, the primary function of the small intestine is nutrient absorption, while the large intestine is responsible for water absorption^13^; to optimally perform these tasks, a gradient in transporter expression exists across the GI tract. It remains unclear if biological differences in the small and large intestine, such as transporter expression, metabolism, or cell type composition, cause drugs to exhibit differential toxicity to the small and large intestine. Current in vitro intestinal models and in vivo histological studies tend to be limited to one part of the GI tract, which prevents differential toxicity from being identified.

Here, we built a scalable murine intestinal monolayer system to provide assessment of toxicity to both the small and large intestines. To help maintain in vivo properties essential for modeling drug-induced toxicity, these intestinal monolayers are derived directly from freshly harvested murine crypts. As a proof-of-concept, we screened 48 clinically used oncology drugs for both small and large intestinal toxicity, revealing that many oncology drugs display differential toxicity across the murine GI tract.

## Results

### Characterizing gastrointestinal toxicity utilizing intestinal monolayers

**Fig. 1:**
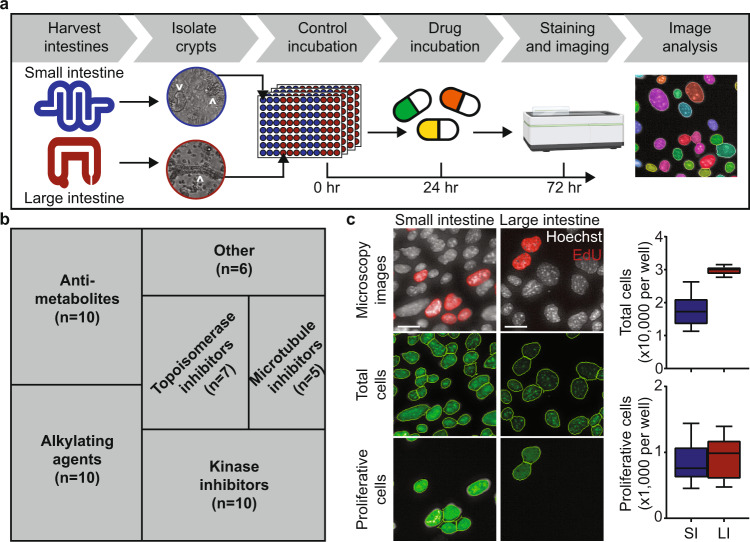
Experimental setup to screen small and large intestine-derived monolayers for drug toxicity. **a** Workflow for culturing and characterizing the effect of oncology drugs in small and large intestine-derived monolayers. Crypts isolated from harvested murine small and large intestines (white arrowheads) are cultured as intestinal monolayers. Intestinal monolayers are grown in control media for 24 h, followed by drug incubation for 48 h. Total and proliferative cell numbers are measured from images of stained intestinal monolayers. Microscope cartoon was created with BioRender.com. **b** Tree plot of the drug classes included in the drug panel. n: number of drugs per class. **c** Images of small and large intestine-derived monolayers grown in control media for 72 h. Scale bars, 20 µm. The total number of cells per well were determined via nuclei segmentation (Hoechst stain) and the number of proliferative cells per well were determined via EdU+ nuclei segmentation. Boxplot showing median value, whiskers showing lower 10th and upper 90th percentiles. *n* = 72 wells. SI: small intestine; LI: large intestine.

We had three main considerations for our toxicity screen (Fig. 1a): drug set, experimental model, and toxicity readout. For our drug panel, we focused on clinically used oncology drugs, which are known to induce widespread GI toxicity^14–17^. We selected 48 FDA-approved oncology drugs that encompass multiple drug classes and diverse treatment indications (Fig. 1b, Supplementary Fig. 1, Supplementary Table 1). We chose two concentrations for each drug: a high concentration (typically greater than the reported cellular IC_50_, Supplementary Table 1) and a low concentration (100-fold lower than the high concentration)^18^. We note that the selected low concentration for most drugs is lower than its clinically observed maximum plasma concentration (*C*_max_, Supplementary Table 1)^19^.

For our experimental model, we chose to make use of monolayers derived from both the murine small and large intestine. First, intestinal monolayers are 2-dimensional and thus are amenable to quantitative high-throughput microscopy^20^. This allowed us to survey the effects of many drugs on both the small and large intestine. Second, intestinal monolayers recapitulate many properties of the intestinal epithelium^21,22^. These properties include apical-basolateral polarization, presence of the major differentiated cell types, cell-cell junctions, and continuous self-renewal (Supplementary Fig. 2a, b)^23–25^. Third, intestinal monolayers recapitulate specific properties of the organ they are derived from. Large intestine-derived monolayers have a greater proportion of goblet (Muc2+) cells, lack Paneth (Lyz+) cells, and have nuclear expression of special AT-rich sequence-binding protein 2 (SATB2), while small intestine-derived monolayers possess Paneth cells and an increased proportion of proliferative (EdU+) cells (Supplementary Fig. 2a, c, d)^26,27^. Fourth, intestinal monolayers are ideal for investigating toxicity intrinsic to the GI epithelium, as they lack mesenchymal cells (Supplementary Fig. 2e)^21^. Fifth, intestine-derived monolayers have similar gene expression to their in vivo counterparts (Supplementary Fig. 3a, b). Finally, intestinal monolayers are derived from primary tissue rather than cancer cell lines. For these reasons, we made use of intestinal monolayers to broadly survey collateral damage of oncology drugs to healthy intestinal epithelial tissue.

To assess cytotoxicity of these oncology drugs to the GI tract, we chose two readouts of tissue health, measuring changes in cell numbers to the whole tissue (differentiated plus active-cycling cells) as well as changes specifically to the proliferative compartment (active-cycling cells only). Intestinal monolayers were cultured in 96-well imaging plates with control media for 24 h, followed by drug incubation for 48 h (Fig. 1a). EdU was incorporated two hours before fixation to label proliferative cells; Hoechst was added after fixation to identify cells’ nuclei. Plates were imaged with an automated confocal microscope and the total number of nuclei and the number of proliferative cells per well were quantified (Fig. 1c). In summary, our image-based screen encompassed: 2 organs (small and large intestine) × 48 oncology drugs × 2 concentrations (100-fold range) × 2 toxicity readouts (total and proliferative cell numbers) × 3 replicates.

### Identification of oncology drugs that differentially target small or large intestine-derived monolayers

**Fig. 2:**
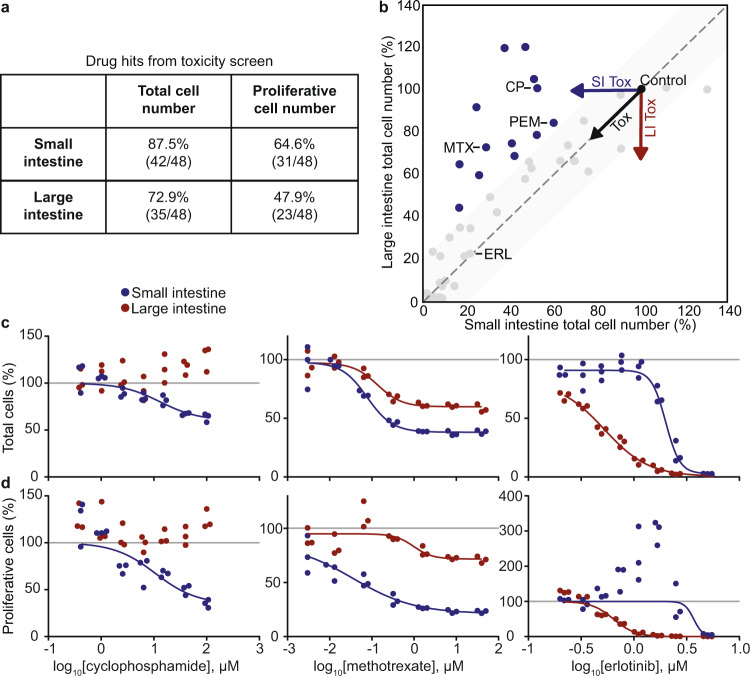
Identification of oncology drugs that differentially target small or large intestine-derived monolayers. **a** Number of drug hits from toxicity screen. **b** Selectivity of the high concentration of each drug in decreasing total cell number. Shaded region represents a selectivity <20%. Each circle is a drug, and blue circles are drugs selective (>20%) for the small intestine. Labeled compounds: MTX, methotrexate; PEM, pemetrexed; CP, cyclophosphamide; ERL, erlotinib. SI: small intestine; LI: large intestine; Tox: toxicity. **c**, **d** Small and large intestine-derived monolayers were treated with a 7–8 point dose-response of cyclophosphamide, methotrexate, and erlotinib, and change in cell number (**c**) or number of proliferative cells (**d**) relative to untreated cells are depicted. *n* = 3 wells. Fitted curves were used to calculate the LC_50_, concentration required to kill 50% of cells, listed in Supplementary Table 3.

As expected, many of the screened oncology drugs decreased both total and proliferative cell numbers in intestinal monolayers (Fig. 2a, Supplementary Fig. 4a, Methods). Overall, small intestine-derived monolayers were generally more sensitive to oncology drugs (Fig. 2b (blue circles), Supplementary Table 2). This is consistent with the observation that small intestine-derived monolayers have an increased proportion of proliferative cells compared to large intestine-derived monolayers (Supplementary Fig. 2d).

Based on our survey, we chose to investigate cyclophosphamide (CP), methotrexate (MTX), and erlotinib (ERL), which were all toxic to the small intestine, but affected the large intestine in varying degrees, ranging from no toxicity, to intermediate toxicity, to strong toxicity, respectively (Fig. 2b, vertical axis). We retested intestinal monolayers across multiple concentrations for these and several mechanistically related drugs. Consistently, small intestine-derived monolayers exhibited dose-dependent toxicity to CP, while large intestine-derived monolayers were completely resistant (Fig. 2c, d, Supplementary Table 3). The antifolates MTX and pemetrexed (PEM) induced stronger toxicity to the proliferative compartment in small intestine compared to large intestine-derived monolayers (Fig. 2d, Supplementary Fig. 4c, Supplementary Table 3). Interestingly, a dose-response of the epidermal growth factor receptor (EGFR) inhibitors erlotinib, gefitinib, and osimertinib revealed selective toxicity to large intestine-derived monolayers at intermediate concentrations (Fig. 2c, d, Supplementary Fig. 4b, c, Supplementary Table 3). In summary, these dose responses revealed that antifolates and CP exhibited increased toxicity to small intestine-derived monolayers, while EGFR inhibitors displayed increased toxicity to large intestine-derived monolayers.

What could account for these differential toxicities? With respect to antifolates and CP, previous work suggests that increased uptake and metabolism (respectively) may be responsible for selective small intestine toxicity^28–31^. For these two cases, we focused on determining if these mechanisms are preserved in intestine-derived monolayers and testing whether differential toxicity is observed in vivo, which has surprisingly not been shown. With respect to EGFR inhibitors, a recent study has observed increased toxicity in the human large intestine^32^. In this case, we focused on elucidating mechanisms of EGFR inhibitor differential toxicity, which are poorly understood.

### Differential antifolate toxicity is due to increased drug uptake in the small intestine

**Fig. 3:**
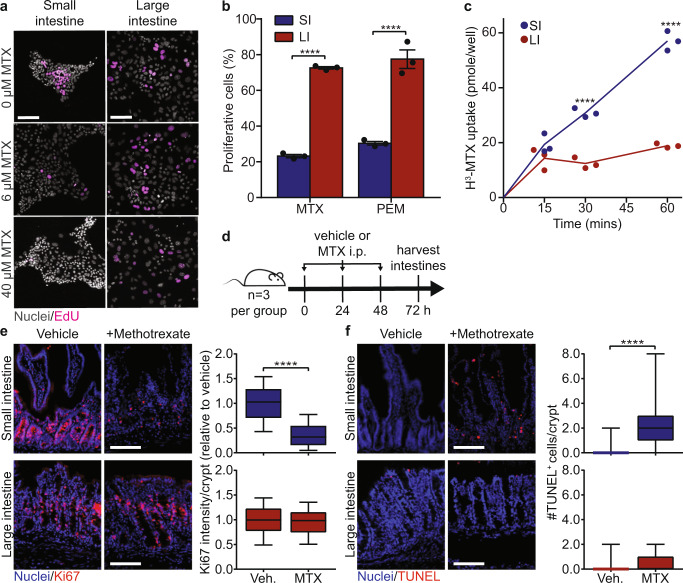
Differential antifolate toxicity is due to increased drug uptake in the small intestine. **a** Representative images of small and large intestine-derived monolayers treated with the indicated methotrexate (MTX) concentration. Nuclei were stained with Hoechst and proliferative cells were stained with EdU. Scale bars, 100 µm. **b** Quantification of the percent change in proliferative cells relative to untreated cells in small and large intestine-derived monolayers treated with 8 µM MTX and 5 µM pemetrexed (PEM) for 48 h. *n* = 3 wells. Statistical significance was calculated by a two-way ANOVA followed by Sidak’s multiple comparison test. **c** Small and large intestine-derived monolayers were incubated with 125 nM tritiated methotrexate (H^3^-MTX) for 15, 30 or 60 min, then the amount of H^3^-MTX per well was measured. *n* = 3 wells. Statistical significance was calculated by an unpaired t-test with Welch’s correction. **d** Schema for MTX in vivo treatment. **e** Changes in proliferation in small and large intestines from mice treated with MTX or vehicle. Representative images of small and large intestines stained for Ki67 and Hoechst. Scale bars, 100 µm. Quantification of the average Ki67 intensity per crypt. Boxplot showing median value, whiskers showing lower 10^th^ and upper 90^th^ percentiles. *n* = 180 crypts from 3 mice (small intestine, blue bars) or *n* = 110 crypts from 3 mice (large intestine, red bars). Statistical significance was calculated by an unpaired *t*-test with Welch’s correction. **f** Changes in apoptosis in small and large intestines from mice treated with MTX or vehicle. Representative images of small and large intestines stained for TUNEL and propidium iodide. Scale bars, 100 µm. Quantification of TUNEL^+^ cells per small (blue bars) and large (red bars) intestinal crypts. Boxplot showing median value, whiskers showing lower 1^st^ and upper 99^th^ percentiles. *n* = 60 crypts from 3 mice. Statistical significance was calculated by an unpaired *t*-test with Welch’s correction. Error bars mean ± SEM. SI: small intestine; LI: large intestine. **** indicates *p*-values < 0.0001.

We investigated whether selective antifolate toxicity towards small intestine-derived monolayers (Fig. 3a, b) is due to differences in antifolate uptake. It has been shown that the intestinal epithelium uptakes MTX and PEM via folate transporters, and that the small intestine exhibits increased folate absorption compared to the large intestine^28,29^. First, we performed an MTX uptake assay in intestinal monolayers and confirmed the small intestine indeed uptakes more tritiated MTX (H^3^-MTX) than the large intestine; MTX uptake plateaued in the large intestine by 15 min but increased roughly linearly for an hour in the small intestine (Fig. 3c). Second, treatment of intestinal monolayers with a potent folate transporter inhibitor, sulfasalazine (SSZ), significantly decreased H^3^-MTX uptake in the small intestine (Supplementary Fig. 5a)^33^. As expected, folate transporters had significantly greater expression in the small intestine compared to the large intestine (reduced folate carrier (RFC) in intestinal monolayers; proton-coupled folate transporter (PCFT) in murine tissue; Supplementary Fig. 5b). Third, we found that the cell-soluble antifolate trimetrexate, which can enter cells without the use of folate transporters^34^, reduced the number of proliferative cells in both small and large intestine-derived monolayers (Supplementary Fig. 5c). Taken together, these data show that small intestine-derived monolayers uptake more MTX, that MTX uptake is folate transporter dependent, and that bypassing transporters eliminates differential toxicity.

We next tested whether MTX selectively targets the small intestine in vivo. Mice were treated with MTX or vehicle (Fig. 3d). After 72 h of treatment, small and large intestines were harvested for histological analysis of proliferation (Ki67) and apoptosis (terminal deoxynucleotidyl transferase (TdT) dUTP nick-end labeling (TUNEL)). RNA was also extracted from small and large intestines, and Ki67 RNA expression was measured by qRT-PCR. MTX treatment significantly decreased Ki67 RNA (Supplementary Fig. 5d) and protein expression (Fig. 3e, Supplementary Fig. 5e) in small intestine crypts, but not in large intestine crypts. MTX treatment also increased the number of apoptotic cells in small intestine crypts, while having no effect on the large intestine (Fig. 3f, Supplementary Fig. 5f). These data confirm that MTX selectively targets the small intestine in vivo.

### Cyclophosphamide-induced small intestinal toxicity is due to increased drug metabolism

**Fig. 4:**
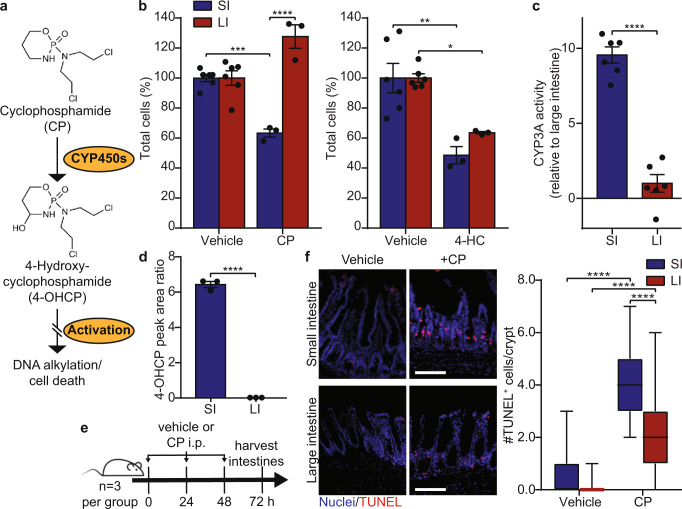
Cyclophosphamide-induced small intestinal toxicity is due to increased drug metabolism. **a** Schematic of cyclophosphamide (CP) activation. **b** Quantification of the percent change in total cells relative to untreated cells in small and large intestine-derived monolayers treated with 100 µM CP and 100 µM 4-hydroperoxycyclophosphamide (4-HC) for 48 h. *n* = 6 wells (control) or *n* = 3 wells (drug treatment). Statistical significance was calculated by a two-way ANOVA followed by Sidak’s multiple comparison test. **c** Measured CYP3A activity in small and large intestine-derived monolayers. *n* = 6 wells. Statistical significance was calculated by an unpaired t-test with Welch’s correction. **d** Detection of 4-hydroxycyclophosphamide (4-OHCP) in the media of small and large intestine-derived monolayers incubated with 100 µM CP for 24 h by LC–MS/MS. Peak area ratio: sample 4-OHCP peak area/4-OHCP internal standard peak area. *n* = 3 wells. Statistical significance was calculated by an unpaired *t*-test with Welch’s correction. **e** Schema for CP in vivo treatment. **f** Changes in apoptosis in small and large intestines from mice treated with CP or vehicle. Representative images of small and large intestines stained for TUNEL and propidium iodide. Scale bars, 100 µm. Quantification of TUNEL^+^ cells per crypt. Boxplot showing median value, whiskers showing lower 1^st^ and upper 99^th^ percentiles. *n* = 60 crypts from 3 mice. Statistical significance was calculated by a two-way ANOVA followed by Sidak’s multiple comparison test. Error bars mean ± SEM. SI: small intestine; LI: large intestine. * indicates *p*-values < 0.05; ** indicates *p*-values < 0.01; *** indicates *p*-values < 0.001; **** indicates *p*-values < 0.0001.

The alkylating agent CP is a pro-drug that requires metabolic activation to 4-hydroxycyclophosphamide (4-OHCP), which then spontaneously breaks down to the active drug phosphoramide mustard (Fig. 4a)^30^. Cytochrome P450s (CYP450) are the main class of enzymes that hydroxylate CP to 4-OHCP (Fig. 4a)^30,35^, and the small intestine expresses multiple CYP450 genes, specifically CYP3A genes^31^. First, we treated intestinal monolayers with 4-hydroperoxycyclophosphamide (4-HC), a stabilized analog of 4-OHCP^36^, to determine if hydroxylated CP causes toxicity to both the small and large intestine. Both CP and 4-HC showed toxicity in the small intestine, while only 4-HC showed toxicity in the large intestine (Fig. 4b, Supplementary Fig. 6a), confirming hydroxylated CP is toxic to both the small and large intestine. Second, we measured greater expression and activity of CYP3A in small intestine compared to large intestine-derived monolayers (Fig. 4c, Supplementary Fig. 6b; increased CYP3A expression is also observed in murine small intestine tissue)^31,37^. In fact, treatment of intestinal monolayers with dexamethasone, a CYP3A transcriptional activator^38^, increased CYP3A activity ~30 fold in the small intestine but showed no induction in the large intestine (Supplementary Fig. 6c). Finally, we detected 4-OHCP by LC-MS/MS only in media collected from small intestine-derived monolayers incubated with CP (Fig. 4d, Supplementary Fig. 6d). These data show that hydroxylated CP (4-HC) causes toxicity to both small and large intestinal monolayers, that small intestinal monolayers have greater CYP3A activity, and that the hydroxylated metabolite (4-OHCP) is only generated in small intestinal monolayers, which indicate that CP-induced small intestinal toxicity is due to metabolism of CP to its active state.

Last, we tested whether CP selectively targets the small intestine in vivo. Mice were treated with CP or vehicle (Fig. 4e). After 72 h of treatment, intestinal tissue and RNA were collected to measure changes to proliferation and apoptosis. CP treatment did not affect proliferation in either the small or large intestine, as shown by a lack of change in Ki67 RNA (Supplementary Fig. 6e). This could be because the small intestine recovered in the 24 h between the last dose of CP and time of tissue harvest. CP treatment did, however, increase the number of apoptotic cells in both small and large intestine crypts, importantly having a more detrimental effect to the small intestine (Fig. 4f, Supplementary Fig. 6f). The detection of low levels of apoptosis in the large intestine in vivo is likely due to the presence of CP metabolites generated in other organs (e.g., the small intestine and liver). These data confirm CP preferentially targets the small intestine in vivo.

### Differential EGFR inhibitor toxicity is due to decreased ERK phosphorylation in the large intestine

**Fig. 5:**
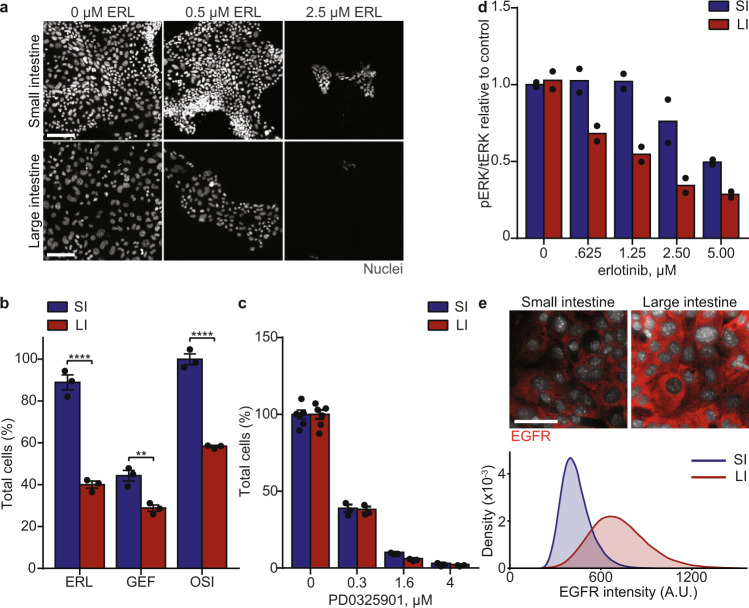
Differential EGFR inhibitor toxicity is due to decreased ERK phosphorylation in the large intestine. **a** Representative images of small and large intestine-derived monolayers treated with indicated erlotinib (ERL) concentration. Nuclei are stained with Hoechst. Scale bars, 100 µm. **b** Quantification of the percent change in total cells relative to untreated cells in small and large intestine-derived monolayers treated with 0.5 µM ERL, 0.8 µM gefitinib (GEF), and 0.4 µM osimertinib (OSI) for 48 h. *n* = 3 wells. Statistical significance was calculated by a two-way ANOVA followed by Sidak’s multiple comparison test. **c** Quantification of the percent change in total cells relative to untreated cells in small and large intestine-derived monolayers treated with the indicated concentration of PD0325901 for 48 h. *n* = 6 wells (vehicle) or *n* = 3 wells (PD0325901). All drug concentrations were statistically significant compared to control (*p*-value < 0.0001) as calculated by a two-way ANOVA followed by Sidak’s multiple comparison test. **d** Quantification of phospho-ERK (pERK) relative to total-ERK (tERK) in small and large intestine-derived monolayers treated with the indicated concentration of erlotinib for 6 h measured by ELISA. *n* = 2 technical replicates. **e** Representative images of EGFR expression in small and large intestine-derived monolayers grown in control media for 24 h. Scale bars, 40 µm. Density plot of EGFR intensity per cell. A.U.: arbitrary units. *n* = >50,000 cells pooled from 5 wells. Error bars mean ± SEM. SI: small intestine; LI: large intestine. ** indicates *p*-values < 0.01; **** indicates *p*-values < 0.0001.

We investigated how EGFR inhibitors selectively target large intestine-derived monolayers (Fig. 5a, b). The epidermal growth factor (EGF) signaling pathway plays a critical role in cell proliferation, as well as maintaining the intestinal stem cell population^39,40^. EGFR signals primarily through the RAS-RAF-MEK-ERK pathway and the PI3K-AKT-mTOR pathway^41^. To determine which of these two pathways are responsible for EGFR inhibitor-induced toxicity, we treated both small and large intestine-derived monolayers with a dose-response of a MEK inhibitor (PD0325901) and an AKT inhibitor (MK2206). MEK inhibition induced toxicity to both the small and large intestine in a dose-dependent manner (Fig. 5c), while AKT inhibition had no effect on either the small or large intestine (Supplementary Fig. 7a). Thus, MEK-ERK signaling is required for survival. To evaluate if ERK is differentially regulated, we measured the ratio of phospho-ERK to total-ERK in both small and large intestine-derived monolayers treated with a dose-response of the EGFR inhibitor erlotinib. These measurements revealed that erlotinib preferentially impairs ERK phosphorylation in the large intestine compared to the small intestine (Fig. 5d).

What mechanisms might underly differential regulation of ERK phosphorylation between the small and large intestine? First, we examined expression of EGFR. Quantification of intestinal monolayers revealed both higher EGFR protein (Fig. 5e) and RNA expression (also in murine intestinal tissue, Supplementary Fig. 7b) in the large intestine compared to small intestine. Second, we measured higher expression of leucine-rich repeats and Ig-like domains-1 (Lrig1), a ligand that acts as an inhibitor of EGFR^42^, in small intestine compared to large intestine-derived monolayers (Supplementary Fig. 7c). Third, we performed an unbiased profiling of phospho-receptor tyrosine kinase activity in small and large intestine-derived monolayers. Interestingly, we identified that the small intestine has increased expression of phospho-human epidermal growth factor receptor 2 (Her2) relative to phospho-EGFR, while these receptors are phosphorylated to similar extents in the large intestine (Supplementary Fig. 7d). This is consistent with prior work, which has shown that Her2 signaling is primarily responsible for maintaining ERK activity in 3-dimensional small intestine organoids^43^. Together, our data reveal that the large intestine is more reliant on EGFR-induced ERK phosphorylation and point to multiple components upstream of ERK that individually or together can lead to its differential regulation in small and large intestine-derived monolayers.

## Discussion

In this study, murine-derived intestinal monolayers provided a scalable system to survey drug-induced GI toxicity and a biologically relevant starting point to investigate mechanisms of toxicity. We identified oncology drugs that cause differential toxicity to the murine small or large intestine and pinpointed biological mechanisms underlying these toxicity differences. Specifically, increased uptake of antifolates in the small intestine led to increased antifolate toxicity, increased metabolism in the small intestine led to increased CP toxicity, and decreased ERK phosphorylation in the large intestine led to increased EGFR inhibitor toxicity. Reassuringly, differential toxicity in intestinal monolayers was predictive of in vivo murine toxicity, as demonstrated by MTX and CP dosing causing increased damage to the murine small intestine.

A natural question is to what degree do murine intestinal models reflect toxicity in human intestines. With respect to EGFR inhibitor-induced differential toxicity, a recent study of non-small cell lung cancer patients treated with erlotinib showed that only the large intestine exhibited signs of erlotinib-induced toxicity^32^. With respect to the mechanisms by which antifolates and CP cause differential toxicity, it is well established that folate absorption and drug metabolism are greater in the human small intestine compared to the large intestine^28–31^. Thus, differential toxicity observed in murine intestinal monolayers may help predict toxicity in human intestines.

There are several limitations of our current investigation. First, our readouts focused on changes to total and proliferative cell numbers but did not examine other possible markers of tissue toxicity, such as inflammation, cell hyperplasia and barrier integrity^44–46^. Second, we focused on acute rather than chronic toxicity, which can be important for medications prescribed over many years, such as NSAIDs^47^. Third, our toxicity screening platform was based only on 2D murine-derived intestinal monolayers. Our study motivates future work to expand cellular readouts of toxicity, as well as to systematically compare 2D vs 3D, and murine- vs human-derived organoid models.

High attrition rates due to drug safety continues to be a key challenge in early drug development^1,2^. Knowing whether a novel therapeutic will induce GI toxicity is essential information for determining if a drug should continue in the development process. Cellular models have been invaluable for assessing pharmacological properties of drug candidates, including permeability and stability. Here, we provide proof-of-concept for using intestinal monolayers derived from fresh murine crypts to study and accurately predict murine GI toxicity of drugs. Moreover, including models of both the small and large intestine revealed biological insights into differences across the gut, enabling better mechanistic understanding of drug-induced GI toxicity.

## Methods

### Mice

All animal care and experimentation were conducted under protocol AN-179937 agreed upon by the Administrative Panel on Laboratory Animal Care at the University of California, San Francisco. All our animal studies are performed in full accordance with UCSF Institutional Animal Care and Use Committee (IACUC). 5- to 6-week-old C57BL/6 mice (C57BL/6NHsd) were purchased from Harlan and housed with ad libitum food and water on a 12 h light cycle at the UCSF Preclinical Therapeutics Core vivarium.

### Intestinal monolayer media

Organoid basal media (OBM) consists of Advanced DMEM/F12 with non-essential amino acids and sodium pyruvate (Fisher Scientific #12634-028) containing 1x N-2 (Fisher Scientific #17502-048), 1x B-27 (Invitrogen #17504-044), 10 mM HEPES (Invitrogen #15630080), 1x GlutaMAX (Invitrogen #35050-061), 1 μM N-acetylcysteine (Sigma Aldrich #A9165), 100 ug/mL Normocin (Invivogen #ant-nr-1), 100 U/mL penicillin and 100 μg/mL streptomycin (Corning #30-002). For initial seeding, intestinal monolayers were maintained in OBM supplemented with 3 μM CHIR-99021 (Sigma Aldrich #SML1046), 50 ng/mL murine EGF (Invitrogen #PMG8043), 1 μM LDN-193189 (Sigma Aldrich #SML0559), 500 ng/mL murine R-spondin-1 (Peprotech #315-32), 100 ng/mL Wnt3a (R&D Systems #5036-WN-500), and 10 μM Y-27632 (Selleck Chemicals #S1049). 4 h after initial seeding, media was changed into WENR media (OBM supplemented with 100 ng/mL Wnt3a, 50 ng/mL murine EGF, 100 ng/mL murine Noggin, and 500 ng/mL murine R-spondin-1). All drugs were applied in the background of WENR media.

### Intestinal monolayer cultures

Small intestine-derived monolayers were cultured from adapted protcols^20–22^. Specifically, jejunum was isolated from male or female mice between 6–10 weeks of age. Epithelium was released from jejunal tissue by incubation in ice-cold PBS with 3 mM EDTA (Ambion #9260) in phosphate buffered saline (PBS, Gibco #10010049). Released epithelial tissue was washed 3x with OBM, after which crypts were separated from villus material using 100 and 70 μm cell strainers (BD Falcon) in succession. Crypts were resuspended in seeding media and plated on Matrigel (Thermo Fisher #CB-40234C)-coated 96-well optical bottom plates (Perkin Elmer #6055302; Greiner #655090).

Large intestine-derived monolayers were cultured by harvesting the large intestine from the same mice described above. The large intestine was first cut open longitudinally, then into 1–2 mm pieces. Large intestine pieces were placed in a falcon tube containing 4 mL of OBM supplemented with 10 μM Y-27632. Then 1.25 mg/mL Collagenase D (Sigma #11088866001), 1.25 U/mL Dispase (STEMCELL Technologies #07913), and 62.5 mU/mL DNAse (STEMCELL Technologies #07900) were added and the falcon tube was placed in a 37 °C incubator for 15 min. After incubation, 5 mM EDTA was added, and the falcon tube was placed in a 37 °C incubator for a second 15 min incubation. Large intestine crypts were separated from epithelial debris using a 100 μm filter. Crypts were washed 1x with OBM, then resuspend in seeding media and plated on Matrigel-coated 96-well optical bottom plates.

For both the small intestine and large intestine, 300 crypts were seeded per well. 4 h after seeding, cells were washed with OBM and incubated in WENR media for 24 h. After WENR media incubation, cells were washed with OBM and incubated with WENR media containing drugs of interest for indicated time.

### Methotrexate and cyclophosphamide administration to mice and tissue harvest

To test for increased toxicity to the small intestine in vivo, MTX (Cayman Chemicals #13960) or CP (Sigma–Aldrich #C3250000) at 100 mg/kg in PBS were administered to mice by intraperitoneal injection at 0, 24, and 48 h. At 72 h, the small and large intestines were harvested for sectioning and intestinal crypts were isolated as described in ‘Intestinal monolayer cultures’. Crypts were lysed in Buffer RLT (RNEasy Kit, Qiagen #74134) for RNA purification.

### Methotrexate uptake assay

To measure uptake of tritiated MTX (H^3^-MTX; American Radiolabeled Chemicals #ART0168), small and large intestine-derived monolayers were cultured in Matrigel-coated 48-well tissue culture plates (Corning #353296) for 48 h. After 48 h, intestinal monolayers were washed with warm OBM and imaged on the IncuCyte S3 automated imaging system (Essen Biosciences) to calculate the confluence of each well. Intestinal monolayers were then incubated with 250 μL WENR media containing 125 nM H^3^-MTX for the indicated times at 37 °C. For inhibition studies, intestinal monolayers were incubated with 250 μL of 125 nM H^3^-MTX in the presence of 500 µM sulfasalazine (SSZ, Cayman Chemicals #15025).

After incubations, intestinal monolayers were washed 3x with 500 μL ice-cold PBS. Intestinal monolayers were then incubated in 300 μL RIPA buffer (Sigma–Aldrich #R0278) for up to 90 min. Then 250 μL of cell lysate was used to measure the amount of H^3^-MTX. 2.5 mL of Ecolite Liquid Scintillation Cocktail (MP Biomedicals #0188247501) was added, and the radioactivity was measured by liquid scintillation counting on a Beckman LS6500 liquid scintillation counter (Beckman Coulter). The average radioactivity from Matrigel-coated only wells was subtracted from all intestinal monolayer wells. Further, radioactivity measurements were normalized to the average confluence of all small intestine or large intestine-derived monolayer wells. Picomoles of H^3^-MTX in each well was calculated by normalizing to a measurement containing 2 μL of 125 nM H^3^-MTX.

### Measuring phospho-ERK and total-ERK

To measure relative levels of phospho-ERK (pERK) compared to total-ERK (tERK), we used an enzyme-linked immunosorbent assay ((ELISA), Abcam #176660). Small and large intestine-derived monolayers were cultured in Matrigel-coated 48-well tissue culture plates for 48 h. After 48 h, intestinal monolayers were washed with warm OBM and incubated with indicated concentration of erlotinib for 6 h. After drug incubation, intestinal monolayers were washed 2× with 250 μL ice-cold PBS and lysed for 30 min. Cell lysates were used to quantify the levels of pERK and tERK according to the manufacturer’s protocol. Measured pERK and tERK levels were first normalized by subtracting background intensity, then each sample’s pERK intensity was normalized to its tERK intensity. Each sample was measured in duplicate.

### Murine phospho-RTK array

The phospho-RTK array was performed according to the manufacturer’s protocol (R&D Systems #ARY014) with cell lysates collected from small and large intestine-derived monolayers cultured for 48 h. Two different biological assays were performed, images are representative of both experiments and quantifications include both experiments.

### CYP3A activity assay

To measure CYP3A activity, the P450-Glo CYP3A4 assay with luciferin-IPA (Promega #V9001) was used^48^. We note CYP3A11 is the murine homolog of CYP3A4^37^. Intestinal monolayers were cultured in 96-well imaging plates with WENR media. For induction assay, WENR media containing dexamethasone was added 4 h after seeding. After 48 h, cells were washed with OBM and WENR media containing 3 μM luciferin-IPA was added. Cells were incubated at 37 °C for one hour. After incubation, cleaved luciferin-IPA was detected by aliquoting supernatant and luciferin detection reagent at a 1:1 ratio to an opaque white 96-well plate (Corning #353296). The plate was incubated at room temperature for 20 min, then luminescence was measured on a Biotek H4 plate reader with an integration time of 1 s/well. CYP3A activity was calculated by first subtracting background luminescence measured from wells containing no cells. Then each well was normalized to its cell viability (see ‘Cell viability assay’).

### Cell viability assay

To measure cell viability, the CellTiter-Glo 3D cell viability assay (Promega #G9681) was used. Intestinal monolayers were cultured and at the indicated time-point an equal amount of CellTiter Glo was added to cells. Plates were put on a shaker for 5 min, then incubated at room temperature for 25 min. After incubation, 100 μL supernatant was transferred to a white opaque 96-well plate, then luminescence was measured on a Biotek H4 plate reader with an integration time of 1 s/well.

### Liquid chromatography tandem mass spectrometry assay

CP and 4-OHCP detection were performed by culturing small and large intestine-derived monolayers in 48-well tissue culture plates for 24 h. After 24 h, intestinal monolayers were washed with OBM and WENR media containing 100 μM CP was added for 24 h. After drug incubation, 100 μL supernatant was collected and 10 μL of 2 M semicarbazide (SCZ) in 50 mM phosphate buffer (pH7.4) was added to convert 4-OHCP to a more stable semicarbazone derivative. Samples were centrifuged at 16,000 *g* for 20 min at 4 °C before using a liquid chromatography tandem mass spectrometry (LC-MS/MS; Shimadzu 20AD XR UFLC pumps and Sciex API5000 tandem mass spectrometer) to detect CP and 4-OHCP.

In brief, 10 μL of samples and internal standards (200 ng/mL CP-d4 and 200 ng/mL 4-OHCP-d4 semicarbazone) were loaded into an oasis HLB 96-well µ-elution solid phase extraction plate. Samples were washed 2x with 50 µL water, eluted 2x with 15 µL acetonitrile, and mixed with 70 µL water. 2 μL of processed samples were injected into a poroshell 120 pentafluorophenyl (PFP) column (50 × 2.1 mm, 2.7 µm, Agilent Tech.), eluted with 10 mM ammonium formate at pH 4 (A) and 0.1% formic acid in acetonitrile (B) in gradient mode [B% (t, min): 4-4-47-90-90-4-4 (0-1-3-3.01-3.50-3.51-4.5)], flow rate was 0.6 mL/min). Electrospray ionization in positive mode and multiple reaction monitoring were used. The ion pairs *m/z* 261→233 for CP, *m/z* 267→237 for CP-d4, *m/z* 334→221 for 4-OHCP-SCZ and *m/z* 340→114 for the internal standard 4-OHCP-d4-SCZ were selected for quantification. MS parameters: CAD, 11; CUR, 20; GS1, 50; GS2, 45; IS, 2000v; TEM: 600 °C; Resolution, high for Q1 and Q3. The retention times were typically 2.80 min for CP and its internal standard and 2.45 min for 4-OHCP and its internal standard. Total run time was 4.5 min per sample. Due to instability of 4-OHCP, the stock solution for calibration curve was generated from 4-hydroperoxycyclophosphamide assuming 100% conversion, followed by in situ derivatization with 2 M SCZ in 50 mM phosphate buffer (pH7.4). Calibration range was 0.19–47.9 µM for CP and 0.070–17.5 µM for 4-OHCP.

### Immunofluorescence assay

Intestinal monolayers were washed 1x with warm D-PBS and then fixed with 4% paraformaldehyde in PBS for 15 min at room temperature. Cells were then washed with PBS and permeabilized with 0.5% Triton-X-100 in PBS at room temperature for 10 min. Cells were washed, blocked with 3% BSA in PBS for 30 min, and then incubated in primary antibody in antibody buffer (PBS with 0.3% Triton-X-100, 1% BSA) overnight at 4 °C. The next day, cells were washed and incubated with secondary antibodies and Hoechst 33342 (5 μg/mL; Invitrogen #H3570) in antibody buffer for 2 h at room temperature.

For histology, intestines were harvested from mice, cut open longitudinally, and incubated in 4% paraformaldehyde in PBS for 2 h at 4 °C. Tissues were then embedded in OCT, frozen, and sectioned at 10 μm. For Ki67 staining, sections were blocked in blocking buffer (0.1 M Tris-HCl, 0.15 M NaCl, 5 µg/mL blocking reagent (Perkin Elmer #FP1020), pH 7.5) containing 5% goat serum (Jackson Labs #005-000-121) for 1 h at room temperature. Sections were than incubated in primary antibody in blocking buffer for 1 h at room temperature. Sections were washed, then incubated with secondary antibody and Hoechst 33342 in blocking buffer for 40 min at room temperature. For TUNEL assays the FITC-TUNEL Assay Kit (Abcam #66108) was used according to the manufacturer’s instructions. Sections were then mounted in Vectashield (Vector Laboratories #H-100) and visualized on the 10x objective of a Nikon Ti Eclipse microscope.

### Antibodies

All antibodies were purchased from suppliers and used as designated without further purification. Antibodies were used as follows:

**Table:** 

Epitope	Vendor and Catalog #	Dilution
Lyz	Dako #A0099	1:2000
ZO-1	Invitrogen #33-9100	1:1000
E-Cadherin	Cell Signaling #3195 S	1:400
SATB2	Santa Cruz Biotechnology #81376	1:50
Villin	BD Biosciences #610358	1:100
Muc2	Santa Cruz Biotechnology #15334	1:100
ChgA	Santa Cruz Biotechnology #393941	1:100
αSMA	Abcam #32575	1:500
Ki67	Cell Signaling #9129 S	1:500
EGFR	Abcam #52894	1:500
Lrig1	R&D Systems #AF3688-SP	1:20

### EdU pulse and visualization

To visualize proliferating cells (specifically, those in S phase), intestinal monolayers were incubated with 10 μM EdU (Thermo Fisher #A10044) in media for 2 h prior to fixation. After immunofluorescence staining, EdU+ cells were visualized using Click chemistry^49^. Cells were incubated with a reaction mixture containing 1 mM CuSO4 (VWR International #470300-880), 5 μM sulfo-Cyanine5 azide (Lumiprobe #B3330) or 5 μM BDP-FL azide (Lumiprobe #11430), and 100 mM sodium ascorbate (Sigma Aldrich #A4034) in PBS for 30 min at room temperature.

### Automated confocal microscopy

Intestinal monolayers were imaged on the 20x water objective of an Operetta CLS High-Content Analysis System on confocal mode with a binning of 2. The area of each well was covered by 61 individual scans. In each field of view, 4 z-planes were collected. Analyzed and representative images were all from maximum projections.

### Immunofluorescence image segmentation and quantification

#### General information

Image segmentation was performed using the PerkinElmer Harmony 4.9 software. Starting with maximum intensity projections of stain images, we segmented and then quantified the number of nuclei, proportion of specific cell types, or stain intensity. The segmentation process for each object type typically consisted of three steps: a preprocessing step, a segmentation step to generate boundaries of objects, and a selection step to select correctly segmented objects.

#### Segmenting nuclei

Hoechst stain images were first smoothed through convolution with a gaussian filter (Width: 3 px). Nuclei were then found using a modified “Find Nuclei” algorithm with Method M (Diameter: 22 μm, Splitting Sensitivity: 0.40, Common Threshold: 0.10). To remove incorrectly segmented nuclei, morphological and intensity properties of each segmented nuclei were calculated. The “Calculate Intensity Properties” algorithm with Method Standard was used to calculate the mean intensity of each segmented nuclei. The “Calculate Morphology Properties” algorithm with Method Standard was used to calculate the roundness of each segmented nuclei. Selected nuclei were found with the “Select Population” algorithm with Method Filter by Property, such that selected nuclei have an intensity > 500 and a roundness > 0.75.

#### Segmenting proliferative cells

EdU stain images were segmented the same as nuclei. The only difference is selected EdU+ nuclei were found using an intensity > 225.

#### Segmenting enteroendocrine cells

Chromogranin A (ChgA) stain images were filtered with the same filter described for nuclei segmentation. Then enteroendocrine cells were found using the “Find Cells” algorithm with Method C (Common Threshold: 0.80, Area: >100 μm^2^, Splitting Coefficient: 200, Individual Threshold: 0.80, Contrast: >0.20). The “Calculate Intensity Properties” algorithm with Method Standard was used to calculate the mean intensity of each segmented enteroendocrine cell. The “Calculate Morphology Properties” algorithm with Method Standard was used to calculate the area and roundness of each segmented enteroendocrine cell. Selected enteroendocrine cells were found with the “Select Population” algorithm with Method Filter by Property, such that each selected enteroendocrine cell has an intensity > 1200, area < 400, and roundness > 0.65.

#### Segmenting goblet cells

Mucin 2 (Muc2) stain images were filtered with the same filter described for nuclei segmentation. Then goblet cells were found using the “Find Cells” algorithm with Method M (Common Threshold: 0.50, Diameter: 25 μm, Splitting Sensitivity: 0.05). The “Calculate Intensity Properties” algorithm with Method Standard was used to calculate the mean intensity of each segmented goblet cell. The “Calculate Morphology Properties” algorithm with Method Standard was used to calculate the area of each segmented goblet cell. Selected goblet cells were found with the “Select Population” algorithm with Method Filter by Property, such that each selected goblet cell has an intensity > 500 and area > 150.

#### Segmenting Paneth cells

Lysozyme (Lyz) stain images were filtered with the same filter described for nuclei segmentation. Then Paneth cells were found and selected using the “Find Cells” algorithm with Method C (Common Threshold: 0.90, Area > 100 μm^2^, Splitting Coefficient: 22.0, Individual Threshold: 0.70, Contrast > 0.10). No selection step was used.

#### Segmenting enterocytes

Villin (Vil) stained regions of images were found using the “Find Image Region” algorithm with Method Common Threshold (Threshold: 0.50, Split into Objects: selected, Area > 20 μm^2^). Nuclei within the Vil+ region were found as described for nuclei segmentation. The “Calculate Intensity Properties” algorithm with Method Standard was used to calculate the mean intensity of the Vil channel in each segmented nucleus. The “Calculate Morphology Properties” algorithm with Method Standard was used to calculate the roundness of each segmented nuclei. Selected nuclei within Vil+ regions were found with the “Select Population” algorithm with Method Filter by Property, such that selected nuclei have a Vil intensity > 500 and roundness > 0.75.

#### Segmenting SATB2+ nuclei

Special AT-rich sequence-binding protein 2 (SATB2) stain images were filtered with the same filter described for nuclei segmentation. SATB2 + nuclei were then found using a modified “Find Nuclei” algorithm with Method M (Diameter: 26 μm, Splitting Sensitivity: 0.30, Common Threshold: 0.20). The “Calculate Intensity Properties” algorithm with Method Standard was used to calculate the mean intensity of the SATB2 and Hoechst channel in each segmented nucleus. The “Calculate Morphology Properties” algorithm with Method Standard was used to calculate the roundness and area of each segmented nucleus. Selected SATB2 + nuclei were found with the “Select Population” algorithm with Method Filter by Property, such that selected SATB2 + nuclei have DAPI intensity >500, 500< SATB2 intensity < 1600, roundness > 0.85, and 70 µm^2^ < area ≤ 300 µm^2^.

#### Quantifying Lrig1 tissue intensity

Leucine-rich repeats and Ig-like domains-1 (Lrig1) stain images were quantified by first identifying tissue regions in each image. Tissue regions were found by the “Find Image Region” algorithm using the Lrig1 channel with Method Common Threshold (Threshold: 0.50, area > 1000px^2^, and Fill Holes selected). The “Calculate Intensity Properties” algorithm with Method Standard was used to calculate the mean intensity of Lrig1 in selected tissue regions. Next, background (non-tissue) regions were found by the “Find Image Region” algorithm using the Lrig1 channel with Method Absolute Threshold (Lowest Intensity ≥ 0, Highest Intensity ≤ 400, area > 0px^2^, and Fill Holes selected). The “Calculate Intensity Properties” algorithm with Method Standard was used to calculate the mean intensity of Lrig1 in background regions. The Lrig1 tissue and background intensity were averaged across 25 fields/well and then Lrig1 background intensity was subtracted from Lrig1 tissue intensity. The measurements of 5 wells are depicted.

#### Quantifying EGFR cellular intensity

To quantify the intensity of EGFR per cell, both EGFR and Hoechst stain images were used. First, all nuclei were found as described in “*segmenting nuclei*.” Then for each nuclei a cell region was calculated using the “Find Surrounding Region” algorithm with Method C (Common Threshold: 0.10, Individual Threshold: 0.70). The “Calculate Intensity Properties” algorithm with Method Standard was used to calculate the mean intensity of EGFR in each selected cell region. Density plots for the intensity of EGFR per cell are depicted and include ≥50,000 cells from 5 wells.

### qRT-PCR

RNA was harvested from both intestinal monolayers and murine crypts using an RNEasy Plus Mini Kit (Qiagen #74136). Reverse transcription was performed using iScript Reverse Transcription kit (Bio-Rad #1708841). Quantitative PCR was performed using SsoAdvanced Universal SYBR Green Supermix (Bio-Rad #1725272) on a BioRad CFXConnect. Test gene values were normalized to *β-actin* values. RNA levels were determined using the following primers:

**Table:** 

Target mRNA	Forward Primer (5′ to 3′)	Reverse Primer (5′ to 3′)
*RFC*	GGGTGTTGTAGTCTGCGTGT	CACTCCACCTTGCACTACCC
*PCFT*	ATCTACCCGGCCACTCTGAA	AGGAAACTGCTGGAACTCCG
*Ki67*	GTCAGCAAGAGGCAGCAAGGGG	CTGGGTCTTTGCCACTGGCTGG
*EGFR*	TCTTCAAGGATGTGAAGTGTG	TGTACGCTTTCGAACAATGT
*Cyp3a11*	TCACACACACAGTTGTAGGGAGAA	GTCCATCCCTGCTTGTTTGTC
*Cyp3a13*	ACCGGCGGCGCTTTG	ATTCTCAGAGATAGAGATGGCCTTTT
*Cyp3a41*	GGTTGTACCACGGGATGTAGTTATAA	TCTGATGTTCTTAGACACTGCC TTTC
β*-actin*	CGCCACCAGTTCGCCATGGA	TACAGCCCGGGGAGCATCGT

### RNA sequencing analysis

RNA was harvested from either intestinal monolayers cultured for 24 h or from freshly isolated murine crypts using an RNEasy Plus Mini Kit. Library preparation and sequencing were outsourced to Genewiz, Inc. (South Plainfield, NJ). RNA sequencing was performed on the Illumina HiSeq. Paired-end sequencing reads were aligned to the reference genome GRCm38 and annotated to vM25.primary_assembly obtained from gencode (htttps://www.gencodegenes.org/mouse) using STAR v.2.7.9a and featureCounts v.2.0.2^50,51^. The obtained gene count data was normalized by using DESeq2 v.3.13^52^, followed by log1p transform. Hierarchical clustering of 110 intestine marker genes was conducted by calculating the Euclidian distance with the “clustermap” function of the package seaborn v.0.11.1 in Python^7,53^. Next, the average normalized gene counts across three replicates were calculated for each sample for the 110 intestine marker genes. The pairwise Pearson’s correlation across samples was calculated using the “corrcoef” function of the package NumPy v.1.21 in Python.

### Toxicity screen data processing

#### Calculation of change in total cell number

The number of nuclei after drug treatment (average of 3 wells) was divided by the number of nuclei in control treatment (average of 6 wells) from the same plate. A drug was counted as having a “toxic” effect on total cell number only if the mean of its high concentration replicates decreased cell number by more than 2 standard deviations (2σ = .28) of the average number of nuclei in control wells across the 8 screened plates.

#### Calculation of change in proliferative cell number

The number of EdU+ cells after each drug treatment (average of 3 wells) was divided by the number of EdU+ cells in the control treatment (average of 6 wells) from the same plate. A drug was counted as having a “toxic” effect on proliferative cell number only if the mean of its high concentration replicates decreased proliferative cell number by more than 2 standard deviations (2σ = 0.58) of the average number of EdU+ cells in control wells across the 8 screened plates.

### Statistics and reproducibility

To calculate statistical significances, we made use of a two-sided two-way analysis of variance (ANOVA) followed by Sidak’s multiple comparison test or a two-sided unpaired t-test with Welch’s correction, as indicated in figure legends. Individual data points are plotted when *n* ≤10, except for Supplementary Fig. 4a.

Most experiments were performed at least twice, with the exception of the initial drug screen (Fig. 2a, b; Supplementary Fig. 4a; Supplementary Table 2), the in vivo methotrexate and cyclophosphamide treatment (Figs. 3e, f; 4f; Supplementary Fig. 5d–f; Supplementary Fig. 6e, f), cyclophosphamide metabolite detection (Fig. 4d; Supplementary 6d), RNA sequencing (Supplementary Fig. 3), and showed reproducible trends. Sample sizes were based on convention in the field. These sample sizes were sufficient given the robust signal changes measured in the experiments. No data were excluded.

### Reporting summary

Further information on research design is available in the Nature Research Reporting Summary linked to this article.

## Supplementary information

Supplementary Information

Description of Additional Supplementary Files

Supplementary Data 1

Supplementary Data 2

Supplementary Data 3

Reporting Summary

## Data Availability

The RNA sequencing data included in this study are deposited in Gene Expression Omnibus (GEO) with the accession code GSE191018. Source data for graphs in the main figures are provided in Supplementary Data 1. Log1p transformed gene count matrix for 110 intestine marker genes generated from RNA sequencing are provided in Supplementary Data 2. Total and proliferative cell numbers with respect to control treated cells generated from the primary screen are provided in Supplementary Data 3. All other data are available from the corresponding authors on reasonable request.

## Code availability

Automated image analysis to quantify the number of total cells and specific cell types was conducted on the Harmony High-Content Imaging and Analysis Software Version 4.9. For detailed parameters see ‘Methods’. Figures were generated from GraphPad Prism Version 7.1.
