# Supplementary material for: Differential toxicity to murine small and large intestinal epithelium induced by oncology drugs
Source: Commun Biol. 2022 Jan 27;5:99. doi: 10.1038/s42003-022-03048-x (PMC8795448; doi:10.1038/s42003-022-03048-x)
Supplement: Supplementary file 1 — Supplementary Information [file 42003_2022_3048_MOESM1_ESM.pdf]

Supplementary Figure 1

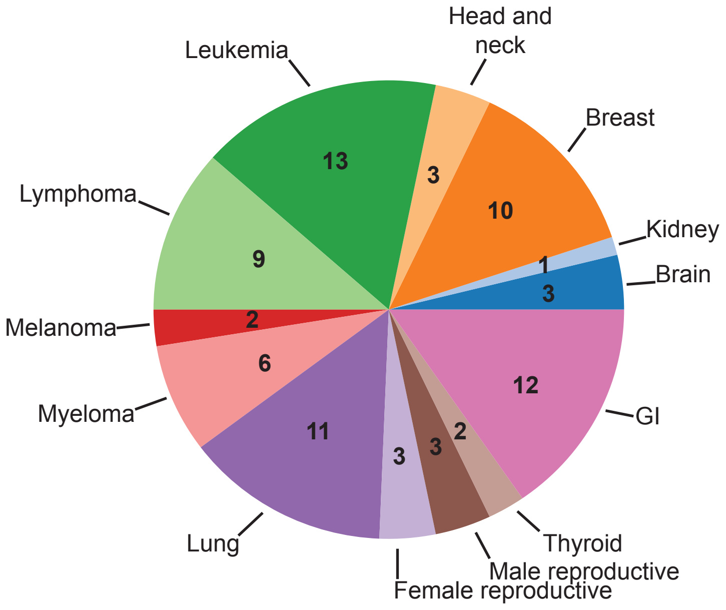

Supplementary Figure 1. Cancer indications encompassed within the drug panel. Pie chart showing the number of drugs in the drug panel used to clinically treat each cancer indication.

Supplementary Figure 2

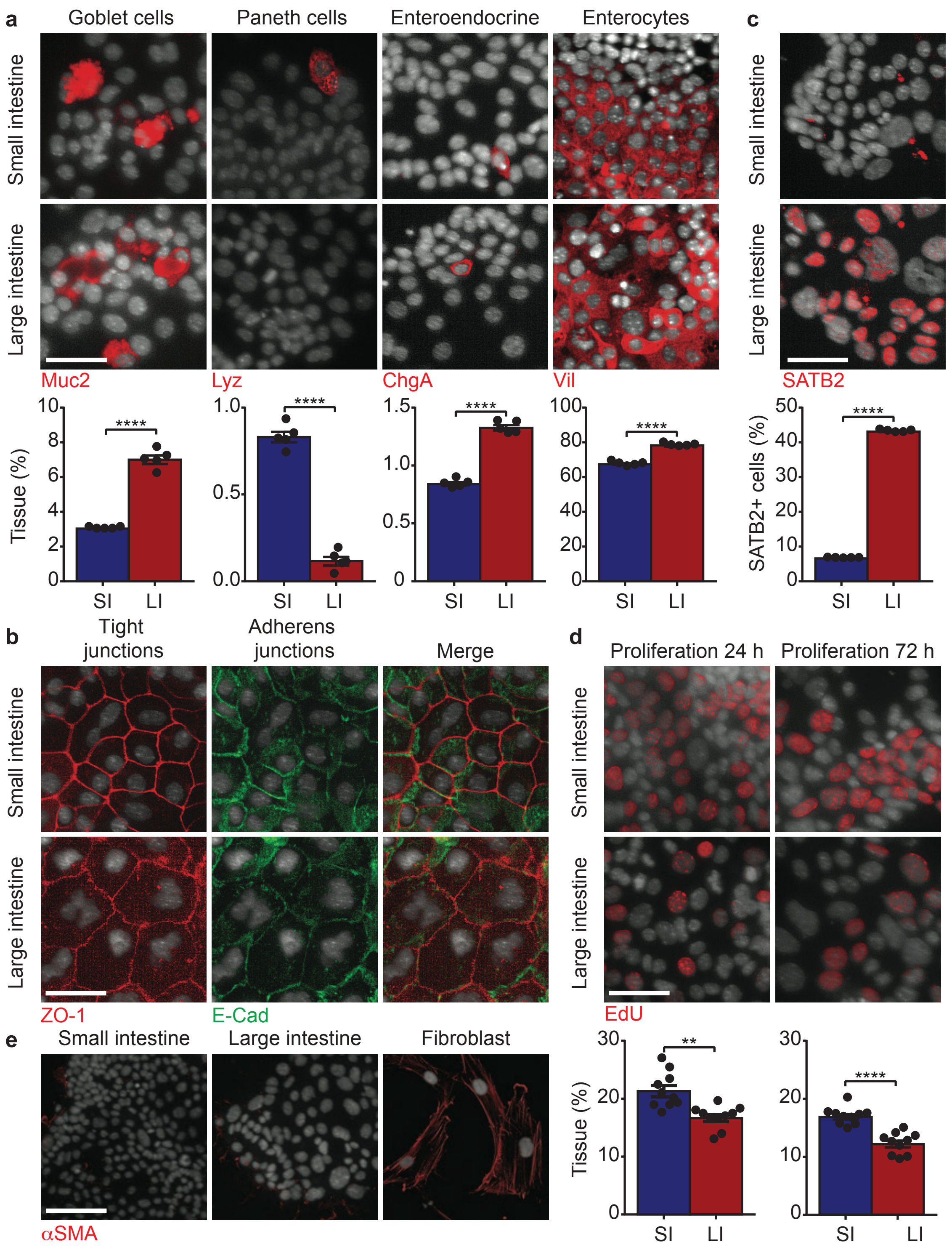

Supplementary Figure 2. Characterization of small and large intestine-derived monolayers.

a. Top: Representative images of cell types in small and large intestine-derived monolayers grown in control media for 24 hours. Nuclei (Hoechst), goblet cells (Muc2), Paneth cells (Lyz), enteroendocrine cells (ChgA), and enterocytes (Vil) are visualized. Scale bars, 40 µm. Bottom: Quantification of the percent of cells in each well that have the indicated cell type marker (#marker+ cells/#total nuclei * 100%). n=5 wells. Statistical significance was calculated by an unpaired t-test with Welch's correction.

b. Representative images of cell-cell junctions in small and large intestine-derived monolayers grown in control media for 72 hours. Nuclei (Hoechst), tight junctions (ZO-1), and adherens junctions (E-Cad) are visualized. Scale bars, 40 µm.

c. Top: Representative images of SATB2+ nuclei in small and large intestine-derived monolayers grown in control media for 24 hours. Scale bars, 40 µm. Bottom: Quantification of the percent of cells that have nuclear SATB2 (#SATB2+ nulclei/#total nuclei * 100%). n=5 wells. Statistical significance was calculated by an unpaired t-test with Welch's correction.

d. Top: Representative images of proliferative cells in small and large intestine-derived monolayers grown in control media for 24 or 72 hours. Scale bars, 40 µm. Bottom: Quantification of the percent of cells that are proliferative (#EdU+ nuclei/#total nuclei * 100%). n=10 wells. Statistical significance was calculated by an unpaired t-test with Welch's correction.

e. Small and large intestine-derived monolayers grown in control media for 48 hours do not display alpha-smooth muscle actin staining (αSMA). Fibroblasts cultures were

stained and imaged in parallel as a positive control for mesenchymal cells (αSMA+).

Scale bars, 80 μm.

Error bars mean +/- SEM. SI: small intestine; LI: large intestine. ** indicates p-values < 0.01; **** indicates p-values < 0.0001.

Supplementary Figure 3

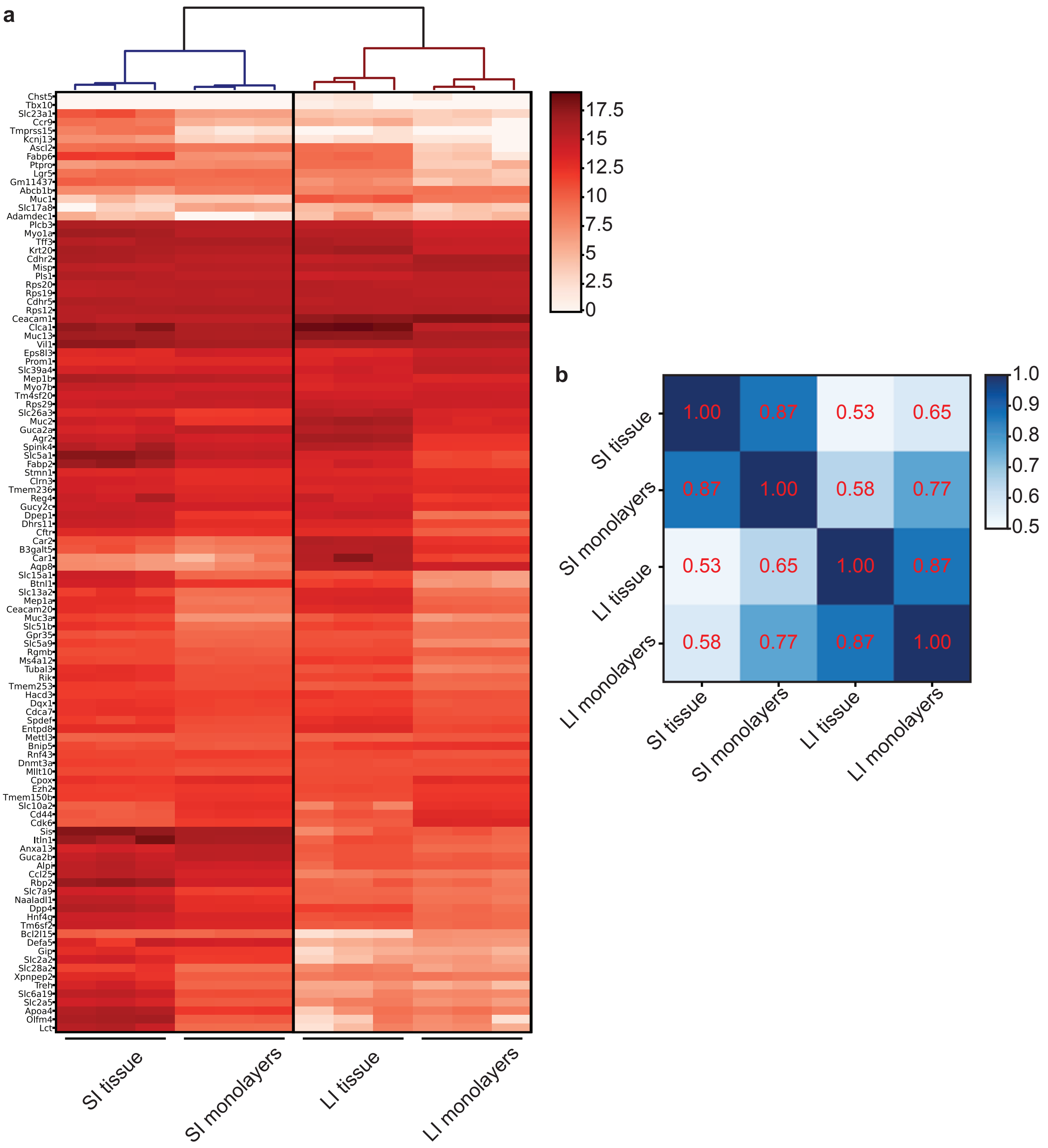

Supplementary Figure 3. Comparison of transcriptome profiles of small and large intestines and cultured intestinal monolayers.

a. Dendrogram (top) and gene expression heatmap (bottom) based on hierarchical clustering of 110 intestine marker genes from RNA sequencing data. RNA was extracted from freshly harvested murine intestines (SI/LI Tissue) or from intestine-derived monolayers (SI/LI Monolayers) grown for 24 hours. Scale bar represents the log transformed range of normalized gene expression. n=3 mice or n=3 pooled wells.

b. Pearson's correlation plot visualizing the correlation (*r*) values for the 110 intestine marker genes between samples. Scale bar represents the range of the correlation coefficients displayed.

Supplementary Figure 4

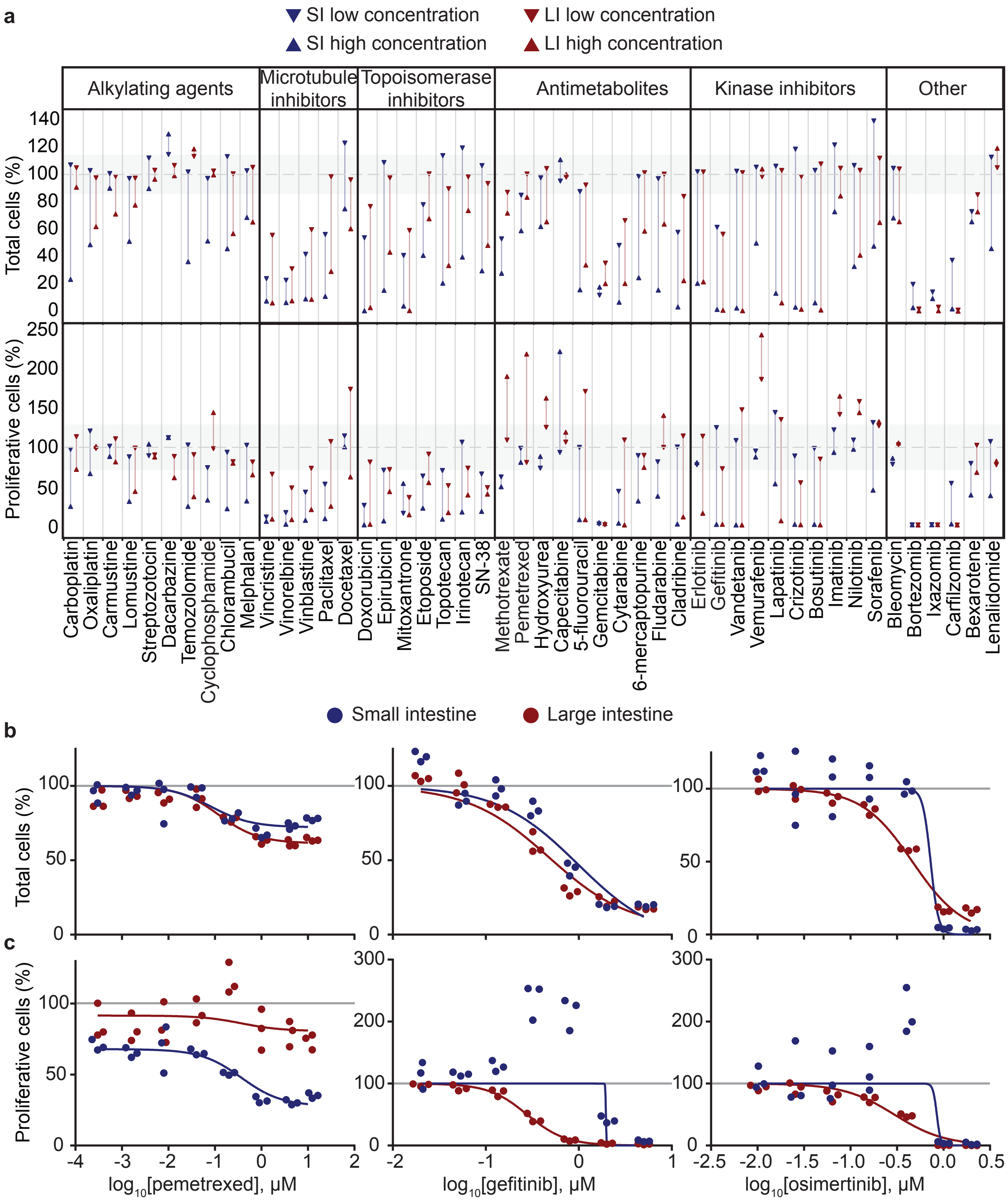

Supplementary Figure 4. Toxicity screen summary and validation of differential toxicity for select drugs.

a. Effect of oncology drugs on total cell number (top) and proliferative cells (bottom) in small and large intestine-derived monolayers. Y-axis shows percent of total or proliferative cells in drug treatment normalized to untreated cells. X-axis shows each drug screened and is sorted by drug class. Blue lines depict small intestine and red lines depict large intestine response. Shaded region is one standard deviation of the plate-to-plate variability between control wells. n=3 wells. SI: small intestine; LI: large intestine.

b,c. Small and large intestine-derived monolayers were treated with a 7-8 point dose-response of pemetrexed, gefitinib, and osimertinib, and (**b**) change in cell number or (**c**) number of proliferative cells relative to untreated cells are depicted. n=3 wells. Fitted curves were used to calculate the LC_{50}, concentration required to kill 50% of cells, shown in Supplementary Table 3.

Supplementary Figure 5

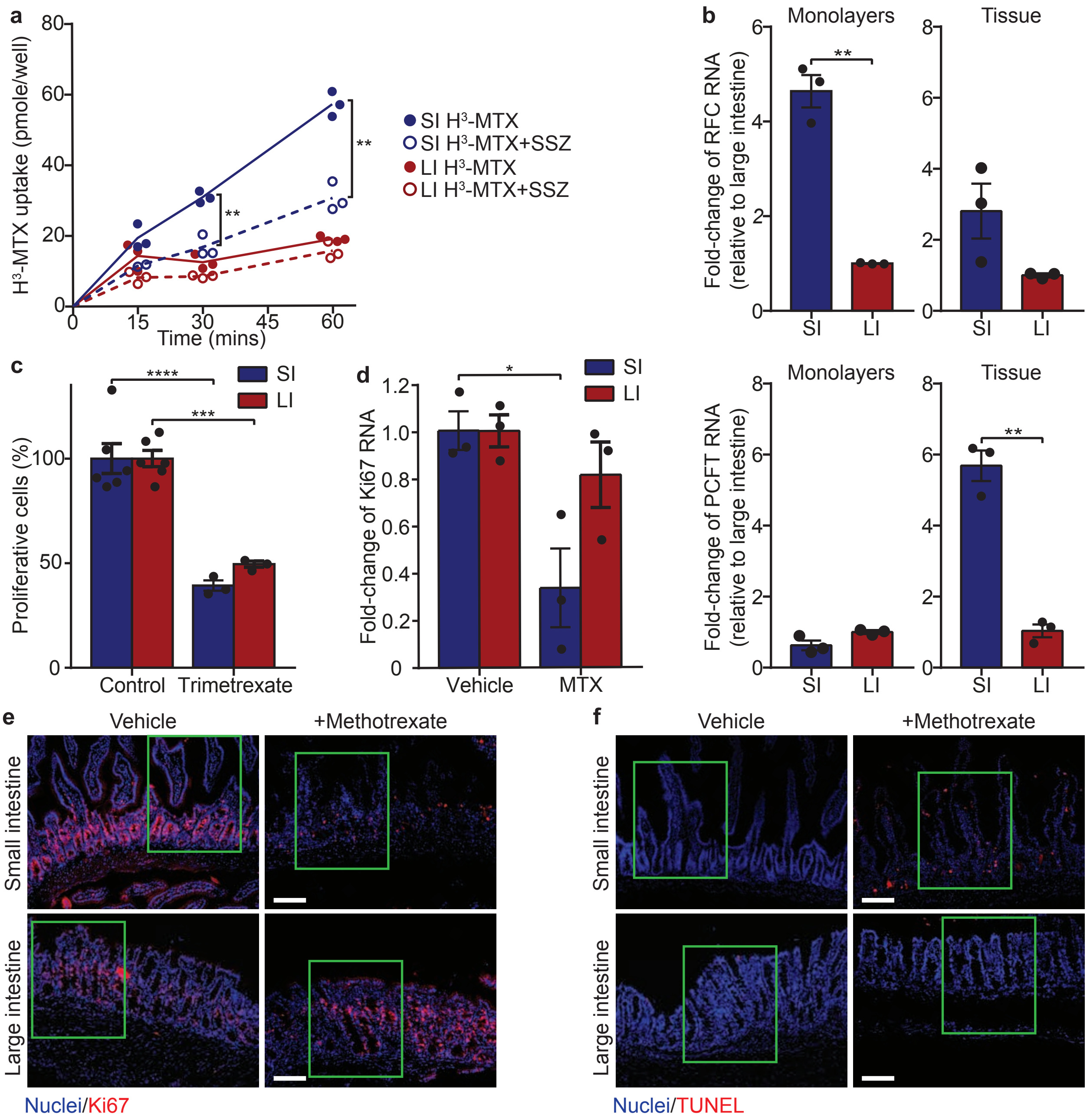

Supplementary Figure 5. Further investigation of differential antifolate toxicity.

a. Small and large intestine-derived monolayers were incubated with 125 nM tritiated methotrexate (H^3-MTX) ± 500 μM sulfasalazine (SSZ) for 15, 30 or 60 minutes, then the amount of H^3-MTX per well was measured. n=3 wells. Statistical significance was calculated by an unpaired t-test with Welch's correction.

b. Folate transporter RNA expression measured by qRT-PCR. Top: Reduced folate carrier (RFC) expression measured in intestinal monolayers cultured for 24 hours (left) and harvested murine intestinal tissue (right). Bottom: Proton-coupled folate transporter (PCFT) expression measured in intestinal monolayers cultured for 24 hours (left) and harvested murine intestinal tissue (right). n=3 pooled samples or n=3 mice. Statistical significance was calculated by an unpaired t-test with Welch's correction.

c. Quantification of the percent change in proliferative cells relative to untreated cells in small and large intestine-derived intestinal monolayers treated with 60 μM trimetrexate for 48 hours. n=6 wells (control) or n=3 wells (trimetrexate). Statistical significance was calculated by a two-way ANOVA followed by Sidak's multiple comparison test.

d. Ki67 RNA expression measured by qRT-PCR. n=3 mice. Statistical significance was calculated by a two-way ANOVA followed by Sidak's multiple comparison test.

e. Zoomed out representative images of small and large intestines stained for Ki67 and Hoechst. Scale bars, 100 μm. Green inset is shown in Fig. 3e.

f. Zoomed out images of small and large intestines stained for TUNEL and propidium iodide. Scale bars, 100 μm. Green inset is shown in Fig. 3f.

Error bars mean +/- SEM. SI: small intestine; LI: large intestine. * indicates p-values < 0.05; ** indicates p-values < 0.01; *** indicates p-values < 0.001; **** indicates p-values < 0.0001.

Supplementary Figure 6

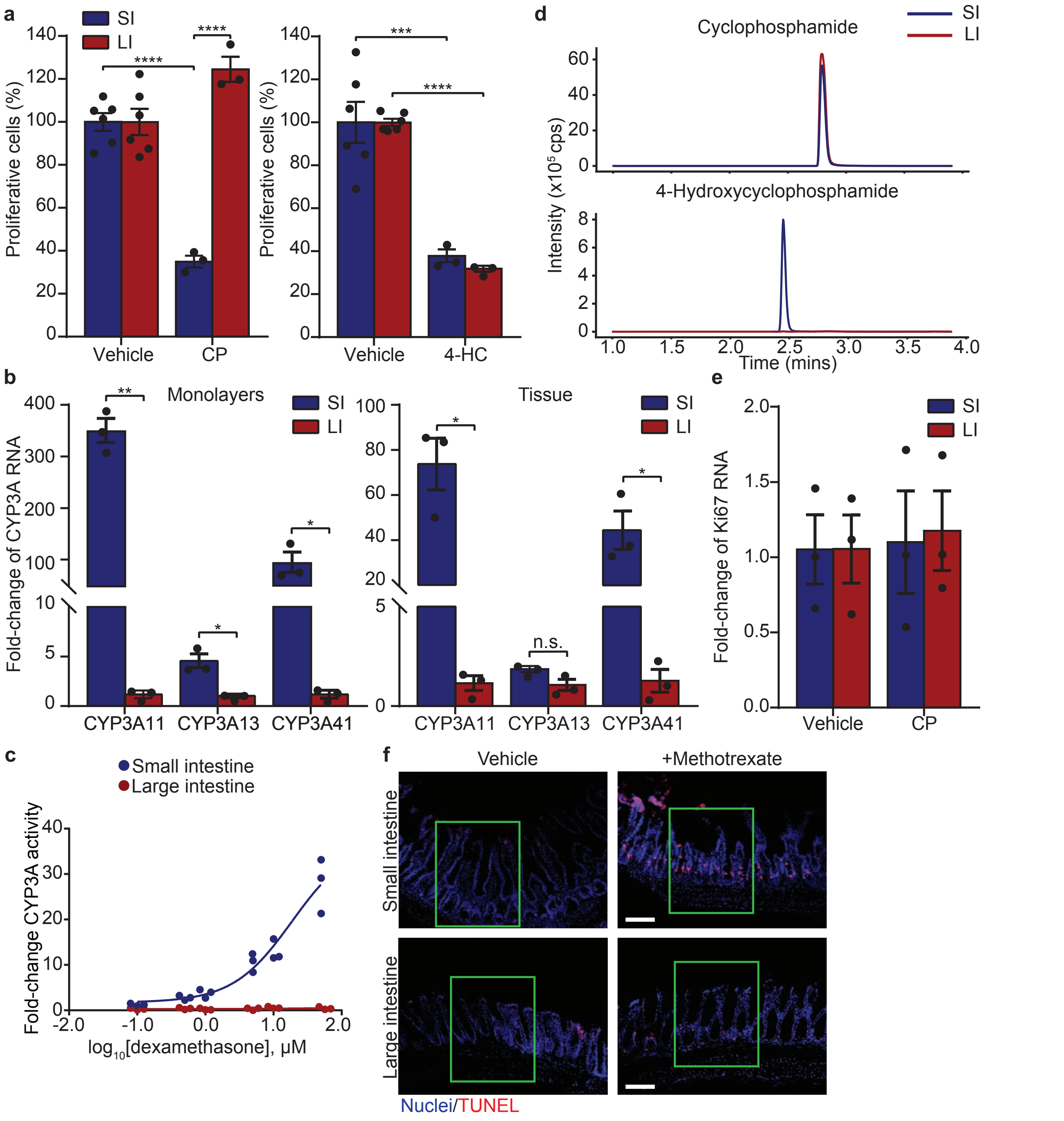

Supplementary Figure 6. Further investigation of differential cyclophosphamide toxicity.

a. Quantification of the percent change in proliferative cells relative to untreated cells in small and large intestine-derived monolayers treated with 100 μM cyclophosphamide (left; CP) and 100 μM 4-hydroperoxycyclophosphamide (right; 4-HC) for 48 hours. n=6 wells (control) or n=3 wells (drug treatment). Statistical significance was calculated by a two-way ANOVA followed by Sidak's multiple comparison test.

b. CYP3A11, CYP3A13, and CYP3A41 RNA expression measured by qRT-PCR. Left: CYP3A RNA expression measured in small and large intestine-derived monolayers cultured for 24 hours. n=3 pooled samples. Right: CYP3A RNA expression measured in harvested murine small and large intestine tissue. n=3 mice. Statistical significance was calculated by an unpaired t-test with Welch's correction.

c. CYP3A activity measured in intestinal monolayers treated with indicated concentration of dexamethasone for 48 hours. Three parameter fit is shown. n=3 wells.

d. Representative chromatogram of cyclophosphamide (top) and 4-hydroxycyclophosphamide (bottom) from LC-MS/MS run.

e. Ki67 RNA expression measured by qRT-PCR. n=3 mice. No statistical significance was calculated by a two-way ANOVA followed by Sidak's multiple comparison test.

f. Zoomed out images of small and large intestines stained for TUNEL and propidium iodide. Scale bars, 100 μm. Green inset is shown in Fig. 4f.

Error bars mean +/- SEM. SI: small intestine; LI: large intestine; n.s.: not significant. * indicates p-values < 0.05;** indicates p-values < 0.01;*** indicates p-values < 0.001; **** indicates p-values < 0.0001.

Supplementary Figure 7

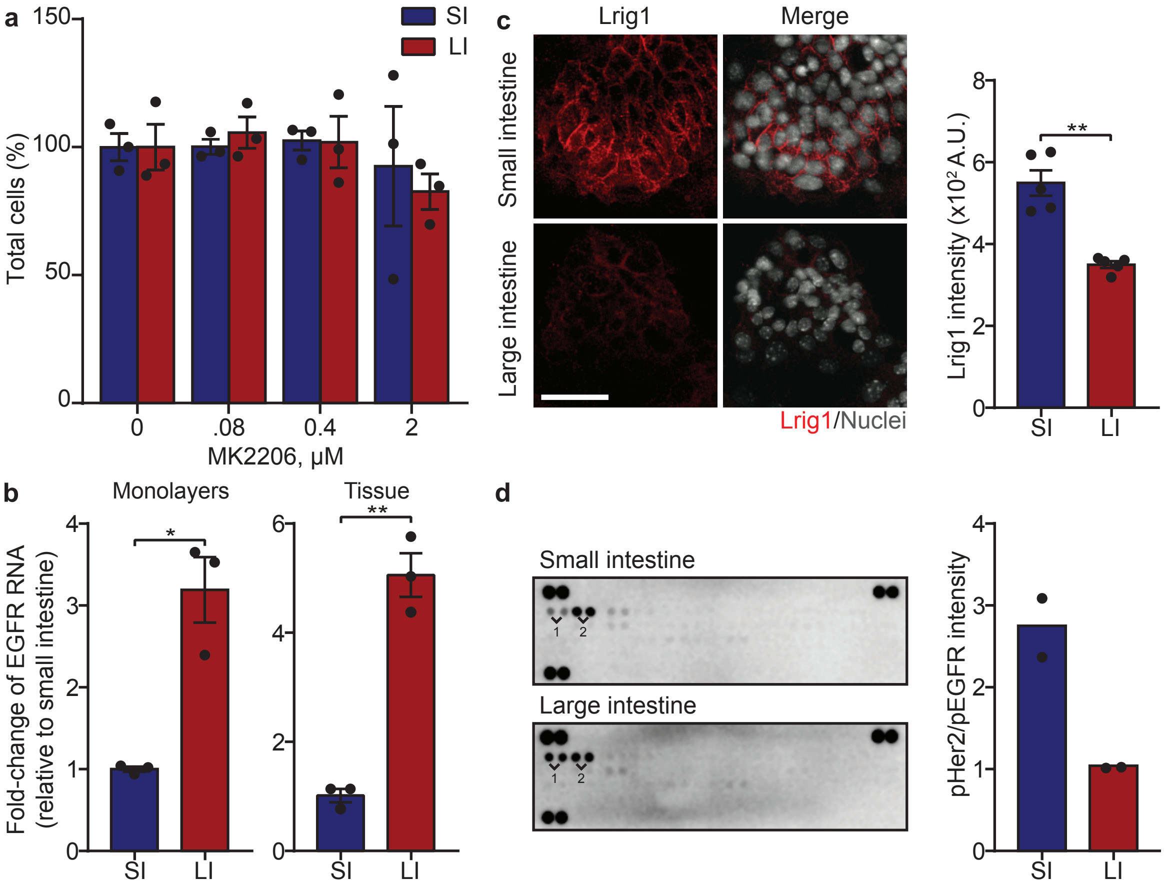

Supplementary Figure 7. Further investigation of differential EGFR inhibitor toxicity.

a. Quantification of the percent change in total cells relative to untreated cells in small and large intestine-derived monolayers treated with the indicated concentration of MK2206 for 48 hours. n=3 wells. No statistical significance between control and drug treated for either the small or large intestine was calculated by a two-way ANOVA followed by Sidak's multiple comparison test.

b. Epidermal growth factor receptor (EGFR) RNA expression measured by qRT-PCR. Left: Expression measured in small and large intestine-derived monolayers cultured for 24 hours. n=3 pooled samples. Right: Expression measured in harvested murine small and large intestine tissue. n=3 mice. Statistical significance was calculated by an unpaired t-test with Welch's correction.

c. Left: Representative images of leucine-rich repeats and Ig-like domains-1 (Lrig1) in small and large intestine-derived monolayers grown in control media for 24 hours. Scale bars, 40 µm. Right: Quantification of Lrig1 intensity in intestinal monolayers. n= 5 wells. A.U.: arbitrary units. Statistical significance was calculated by an unpaired t-test with Welch's correction.

d. Left: Representative images of a mouse phospho-RTK array for small intestine-derived monolayers (top) and large intestine-derived monolayers (bottom). 1: pEGFR; 2: pHer2. Right: Quantification of the relative intensity of phospho-Her2 (pHer2) to phospho-EGFR (pEGFR). n=2 biological replicates.

Error bars mean +/- SEM. SI: small intestine; LI: large intestine; * indicates p-values < 0.05;** indicates p-values < 0.01.

Supplementary Table 1. Screened oncology drugs.

Class	Subclass	Name	Concentrations (µM)	IC50 (µM)[18]	Cmax (µM)[19]	GI Adverse Events*
alkylating agents	metal salts	carboplatin	40, 0.4	na	135.00	v,p,d,c
		oxaliplatin	50, 0.5	41	4.96	v,n,m
	nitrosoureas	carmustine	100, 1	452	19.40	v,n
		lomustine	100, 1	na	na	v,n,s
		streptozocin	10, 0.01	na	1438.00	v,n,d
	hydrazines/ triazines	dacarbazine	2.5, 0.025	na	34.40	v,n
		temozolomide	20, 0.2	375	37.60	v,n,d,c
	nitrogen mustards	cyclophosphamide	100, 1	180	128.00	v,n,d,co
		chlorambucil	100, 1	na	1.62	v,n,d
		melphalan	10, 0.1	na	9.17	v,n,d,m
microtubule inhibitors	destabilizing	vincristine	5, 0.05	0.20	1.48	c
		vinorelbine	40, 0.4	0.05	0.81	v,n,c
		vinblastine	5, 0.05	0.03	0.04	v,n,d,c,m
	stabilizing	paclitaxel	5, 0.05	0.08	4.27	v,n
		docetaxel	100, 1	0.1	5.47	v,n,d,c,co
topoisomerase inhibitors	I	topotecan	40, 0.4	1.1	0.02	v,n,d
		irinotecan	30, 0.3	14	5.78	v,n,p,d,c
		SN-38	5, 0.05	0.02	0.14	v,n,p,d,c
	II	doxorubicin	50, 0.5	0.2	6.73	v,n
		epirubicin	10, 0.01	0.4	16.60	v,n,d,m
		mitoxantrone	90, 0.9	1.6	0.72	v,n,m
		etoposide	50, 0.5	4.7	33.40	p,c
antimetabolite	antifolates	methotrexate	40, 0.4	0.6	1.31	v,d,s
		pemetrexed	5, 0.05	15	306.00	v,n,a
	urea	hydroxyurea	100, 1	na	795.00	unspecified
	pyrimidine analogs	5-fluorouracil	100, 1	110	426.00	d,m
		capecitabine	30, 0.3	na	21.10	v,n,d
	purine analogs	6-mercaptopurine	20, 0.2	na	0.59	n
		fludarabine	50, 0.5	111	na	v,n
		cladribine	30, 0.3	na	0.17	p
	nucleoside analogs	gemcitabine	50, 0.5	0.03	89.30	v,n
		cytarabine	100, 1	4.6	54.40	v,n
kinase inhibitors	EGFR	erlotinib	2.5, 0.025	13	3.15	v,n,d
		gefitinib	100, 1	23	0.36	d
	VEGFR/EGFR	vandetanib	10, 0.1	na	2.16	n,p,d,co
	BRAF	vemurafenib	10, 0.1	na	127.00	n
	HER2	lapatinib	10, 0.1	21	4.18	n,d
	ALK	crizotinib	50, 0.5	21	0.91	v,n,d,c
	BCR/ABL	bosutinib	10, 0.1	5.9	0.38	v,n,p,d
		imatinib	10, 0.1	16	7.50	v,n,p,d
		nilotinib	10, 0.1	30	0.84	n,p,d,c
	multiple	sorafenib	10, 0.1	14	20.10	n,p,d,b
other antineoplastics	antibiotic	bleomycin	5, 0.05	5.4	706.00	none
	proteasome inhibitors	bortezomib	100, 1	0.01	0.31	v,n,d,c
		ixazomib	100, 1	na	0.12	v,n,d,c
		carfilzomib	20, 0.2	na	5.88	none
	retinoid	bexarotene	20, 0.2	32	3.39	n,p
	immunomodulator	lenalidomide	50, 0.5	46	1.74	n,d,c

* Common clinical GI adverse events listed on FDA drug prescription label.
[18]Geometric mean of screened cancer lines in the Genomics of Drug Sensitivity in Cancer database
v: vomiting; p: pain; d: diarrhea; c: constipation; n: nausea; m: mucositis; co: colitis; s: stomatitis; a: anorexia; b: bleeding; na: not available

Supplementary Table 2. Drug hits for total (top) and proliferative (bottom) cell numbers by drug class.

	Alkylating agents	Microtubule inhibitors	Topo. inhibitors	Anti-metabolite	Kinase inhibitors	Other
Small intestine	70% (7/10)	80% (4/5)	100% (7/7)	90% (9/10)	90% (9/10)	100% (6/6)
Large intestine	40% (4/10)	100% (5/5)	86% (6/7)	80% (8/10)	80% (8/10)	67% (4/6)

	Alkylating agents	Microtubule inhibitors	Topo. inhibitors	Anti-metabolite	Kinase inhibitors	Other
Small intestine	60% (6/10)	80% (4/5)	86% (6/7)	60% (6/10)	40% (4/10)	83% (5/6)
Large intestine	10% (1/10)	80% (4/5)	71% (5/7)	40% (4/10)	60% (6/10)	50% (3/6)

Supplementary Table 3. Calculated LC_{50} for drug dose-responses.

	Total cell number		Proliferative cell number	
	Small intestine (μM)	Large intestine (μM)	Small intestine (μM)	Large intestine (μM)
Cyclophosphamide	N.A.	N.A.	30.55	N.A.
Methotrexate	0.202	N.A.	0.053	N.A.
Pemetrexed	N.A.	N.A.	0.298	N.A.
Erlotinib	1.959	0.417	3.733	0.646
Gefitinib	0.885	0.519	1.995	0.282
Osimertinib	0.740	0.471	0.863	0.301

N.A.: not applicable
