# Supplementary material for: Differential toxicity to murine small and large intestinal epithelium induced by oncology drugs
Source: Commun Biol. 2022 Jan 27;5:99. doi: 10.1038/s42003-022-03048-x (PMC8795448; doi:10.1038/s42003-022-03048-x)
Supplement: Supplementary file 2 — Description of Additional Supplementary Files [file 42003_2022_3048_MOESM2_ESM.pdf]

Description of Additional Supplementary Files

File name: Supplementary Data 1
Description: Source data for main figure graphs.

File name: Supplementary Data 2
Description: Gene count matrix of 110 intestine marker genes.

File name: Supplementary Data 3
Description: Processed data from primary drug screen.
