# Supplementary material for: Differential toxicity to murine small and large intestinal epithelium induced by oncology drugs
Source: Commun Biol. 2022 Jan 27;5:99. doi: 10.1038/s42003-022-03048-x (PMC8795448; doi:10.1038/s42003-022-03048-x)
Supplement: Supplementary file 6 — Reporting Summary [file 42003_2022_3048_MOESM6_ESM.pdf]

nature research

Corresponding author(s): Steven Altschuler and Lani Wu

Last updated by author(s): Dec 15, 2021

Reporting Summary

Nature Research wishes to improve the reproducibility of the work that we publish. This form provides structure for consistency and transparency in reporting. For further information on Nature Research policies, see our Editorial Policies and the Editorial Policy Checklist.

Statistics

For all statistical analyses, confirm that the following items are present in the figure legend, table legend, main text, or Methods section.

n/a	Confirmed	
☐	☒	The exact sample size (*n*) for each experimental group/condition, given as a discrete number and unit of measurement
☐	☒	A statement on whether measurements were taken from distinct samples or whether the same sample was measured repeatedly
☐	☒	The statistical test(s) used AND whether they are one- or two-sided<br>*Only common tests should be described solely by name; describe more complex techniques in the Methods section.*
☒	☐	A description of all covariates tested
☐	☒	A description of any assumptions or corrections, such as tests of normality and adjustment for multiple comparisons
☐	☒	A full description of the statistical parameters including central tendency (e.g. means) or other basic estimates (e.g. regression coefficient) AND variation (e.g. standard deviation) or associated estimates of uncertainty (e.g. confidence intervals)
☐	☒	For null hypothesis testing, the test statistic (e.g. *F*, *t*, *r*) with confidence intervals, effect sizes, degrees of freedom and *P* value noted<br>*Give P values as exact values whenever suitable.*
☒	☐	For Bayesian analysis, information on the choice of priors and Markov chain Monte Carlo settings
☒	☐	For hierarchical and complex designs, identification of the appropriate level for tests and full reporting of outcomes
☐	☒	Estimates of effect sizes (e.g. Cohen's *d*, Pearson's *r*), indicating how they were calculated

Our web collection on statistics for biologists contains articles on many of the points above.

Software and code

Policy information about availability of computer code

Data collection	Harmony High-Content Imaging and Analysis Software Version 4.9
Data analysis	Harmony High-Content Imaging and Analysis Software Version 4.9 and GraphPad Prism Version 7.1

For manuscripts utilizing custom algorithms or software that are central to the research but not yet described in published literature, software must be made available to editors and reviewers. We strongly encourage code deposition in a community repository (e.g. GitHub). See the Nature Research guidelines for submitting code & software for further information.

Data

Policy information about availability of data

All manuscripts must include a data availability statement. This statement should provide the following information, where applicable:

- Accession codes, unique identifiers, or web links for publicly available datasets
- A list of figures that have associated raw data
- A description of any restrictions on data availability

The RNA sequencing data included in this study are deposited in Gene Expression Omnibus (GEO) with the accession code GSE191018. Source data for graphs in the main figures are provided in Supplementary Data 1. Log1p transformed gene count matrix for 110 intestine marker genes generated from RNA sequencing are provided in Supplementary Data 2. Total and proliferative cell numbers with respect to control treated cells generated from the primary screen are provided in Supplementary Data 3. All other data are available from the corresponding author on reasonable request.

Field-specific reporting

Please select the one below that is the best fit for your research. If you are not sure, read the appropriate sections before making your selection.

[x] Life sciences [ ] Behavioural & social sciences [ ] Ecological, evolutionary & environmental sciences

For a reference copy of the document with all sections, see nature.com/documents/nr-reporting-summary-flat.pdf

Life sciences study design

All studies must disclose on these points even when the disclosure is negative.

Sample size	Sample sizes were based on convention in the field. These sample sizes were sufficient given the robust signal changes measured in the experiments.
Data exclusions	No data were excluded.
Replication	Most experiments were performed at least twice, with the exception of the initial drug screen (Figure 2A,B; Supplementary Figure 4A; Supplementary Table 2), the in vivo methotrexate and cyclophosphamide treatment (Figure 3E,F; Figure 4F; Supplementary Figure 5D-F; Supplementary Figure 6E,F), cyclophosphamide metabolite detection (Figure 4D; Supplementary 6D), RNA sequencing (Supplementary Figure 3), and showed reproducible trends.
Randomization	Randomization was not required for any of the experiments in this study.
Blinding	Blinding was not required for any of the experiments in this study.

Reporting for specific materials, systems and methods

We require information from authors about some types of materials, experimental systems and methods used in many studies. Here, indicate whether each material, system or method listed is relevant to your study. If you are not sure if a list item applies to your research, read the appropriate section before selecting a response.

Materials & experimental systems

n/a	Involved in the study	
[ ]	[x]	Antibodies
[x]	[ ]	Eukaryotic cell lines
[x]	[ ]	Palaeontology and archaeology
[ ]	[x]	Animals and other organisms
[x]	[ ]	Human research participants
[x]	[ ]	Clinical data
[x]	[ ]	Dual use research of concern

Methods

n/a	Involved in the study	
[x]	[ ]	ChIP-seq
[x]	[ ]	Flow cytometry
[x]	[ ]	MRI-based neuroimaging

Antibodies

Antibodies used	The rabbit polyclonal anti-Lysozyme antibody was purchased from Dako (#A0099). The mouse monoclonal anti-ZO-1 antibody was purchased from Invitrogen (#33-9100). The rabbit monoclonal anti-E-Cadherin antibody and the rabbit monoclonal anti-Ki-67 antibody were purchased from Cell Signaling Technology (#3195S; #9129S). The mouse monoclonal anti-SATB2 antibody, rabbit polyclonal anti-Muc2 antibody, and mouse monoclonal anti-Chr-A were purchased from Santa Cruz Biotechnology (#81376; #15334; #393941). The mouse monoclonal anti-Villin antibody was purchased from BD Biosciences (#610358). The rabbit monoclonal anti-alpha smooth muscle actin antibody and the rabbit monoclonal anti-EGFR antibody were purchased from Abcam (#32575; #52894). The goat polyclonal anti-LRIG1 antibody was purchased from R&D Systems (#AF3688-SP).
Validation	Anti-Lysozyme primary antibody: broadly used antibody with >100 citations, as retrieved from CiteAb. Anti-ZO-1 primary antibody: broadly used antibody with >500 citations, as retrieved from the manufacturer's website. Anti-E-Cadherin primary antibody: broadly used antibody with >1200 citations, as retrieved from the manufacturer's website. Anti-SATB2 primary antibody: antibody with >30 citations, as retrieved from CiteAb. Anti-Villin primary antibody: antibody with >5 citations, as retrieved from CiteAb. Anti-Muc2 primary antibody: broadly used antibody with >200 citations, as retrieved from CiteAb. Anti-Chr-A primary antibody: antibody with >10 citations, as retrieved from CiteAb. Anti-alpha smooth muscle actin primary antibody: broadly used antibody with >300 citations, as retrieved from CiteAb. Anti-Ki-67primary antibody: broadly used antibody with >100 citations, as retrieved from CiteAb. Anti-EGFR primary antibody: broadly used antibody with >200 citations, as retrieved from CiteAb. Anti-LRIG1 primary antibody: broadly used antibody with >300 citations, as retrieved from CiteAb.

Animals and other organisms

Policy information about studies involving animals; ARRIVE guidelines recommended for reporting animal research

Laboratory animals	C57BL/6 female and male mice 6-10 weeks of age were used for all experiments.
Wild animals	This study did not include wild animals.
Field-collected samples	This study did not include samples collected from the field.
Ethics oversight	Administrative Panel on Laboratory Animal Care at the University of California, San Francisco and University of California, San Francisco Institutional Animal Care and Use Committee.

Note that full information on the approval of the study protocol must also be provided in the manuscript.
